# Isocaloric Pair-Fed High-Carbohydrate Diet Induced More Hepatic Steatosis and Inflammation than High-Fat Diet Mediated by miR-34a/SIRT1 Axis in Mice

**DOI:** 10.1038/srep16774

**Published:** 2015-11-26

**Authors:** Xinli Li, Fuzhi Lian, Chun Liu, Kang-Quan Hu, Xiang-Dong Wang

**Affiliations:** 1 Nutrition and Cancer Biology Laboratory, Jean Mayer USDA Human Nutrition Research Center on Aging at Tufts University, Boston, MA, USA, 02111; 2 School of Public Health, Medical College of Soochow University, Suzhou, Jiangsu, China, 215123

## Abstract

To investigate the different effects of isocaloric high-fat diet (HFD) and high-carbohydrate diet (HCD) on hepatic steatosis and the underlying mechanisms, especially the role of microRNA-34a/silent information regulator T1 (SIRT1) axis, C57BL/6J mice (n = 12/group) were isocaloric pair-fed with Lieber-DeCarli liquid diet containing either high fat (HFLD) or high carbohydrate (HCLD) for 16 weeks. As compared to the HFLD fed mice, despite the similar final body weights, HCLD feeding: (1) induced more severe hepatic steatosis; (2) up-regulated hepatic expression of miR-34a accompanied with significant decrease of SIRT1 and nicotinamide phosphoribosyltransferase (NAMPT), SIRT1 activity and phosphorylation of AMPK; (3) up-regulated *de novo* lipogenesis (DNL) related proteins expression (ACC, SCD1), and down-regulated expressions of miR-122, miR-370 and miR-33; (4) decreased mRNA expressions of genes *Cpt1, Pparα and Pgc1α* related to fatty acid oxidation; (5) increased hepatic total cholesterol concentration and decreased expression of cholesterol metabolism related genes *Abcg5, Abcg8, Abcg11, Cyp7a1* and *Cyp8b1*; and (6) induced higher hepatic inflammatory response accompanied with significant increased mRNA expressions of *Il1β*, *Tnfα* and *Mcp1*. Thus, isocaloric HCLD feeding induced greater severity in hepatic steatosis and inflammatory response than HFLD feeding, potentially through miR-34a/SIRT1 axis mediated promotion of DNL, inhibition of fatty acid oxidation and cholesterol metabolism.

Hepatic steatosis in nonalcoholic fatty liver disease (NAFLD) is usually a benign and reversible course, which can also further develop into more detrimental stages, such as nonalcoholic steatohepatitis (NASH), fibrosis, cirrhosis and increase liver cancer risk^1^. The interaction of genetic determinants, nutritional factors and lifestyle play a critical role in the induction of hepatic steatosis. Previous studies had highlighted the effects of high-fat diet (HFD) on the induction of steatosis by regulating energy metabolism^2^,^3^, *de novo* lipogenesis (DNL), translocation of triglycerols (TG), reverse of cholesterol transport and oxidation of free fatty acids in mitochondria^4^. Furthermore, HFD-induced hepatic steatosis could up-regulate the expression of tumor necrosis factor α (TNFα) and inhibit adiponectin signaling, which resulted in increased inflammation in liver tissue^5^ and subsequent transition of steatosis to NASH^6^. Therefore, HFD is regarded as one of the critical dietary factors involved in the initiation of hepatic steatosis, and low-fat and high-carbohydrate diet had been recommended for providing health benefits over the past several years.

On the other hand, both epidemiological and experimental studies had supported the involvement of high carbohydrates diet (HCD) in promoting the development of fatty liver by induction of hepatic DNL and increased synthesis of lipogenic enzymes^7^,^8^,^9^,^10^. Our recent study demonstrated that HCD induced more steatosis than HFD by up-regulating the protein expression of acetyl-CoA carboxylase 1 (ACC) and stearoyl-CoA desaturase 1 (SCD1) in mice^9^. However, in this study, there was no strict control of the energy intake and body weight gain because the mice were fed *ad libitum*. This raised an important question whether HCD could induce more hepatic lesions than HFD fed mice under the isocaloric intake.

Silent information regulator T1 (SIRT1), a NAD^+^-dependent histone/protein deacetylase, is an important regulator of energy metabolism, inflammation and mitochondrial function^11^,^12^,^13^,^14^. SIRT1 has recently been implicated in the regulation of obesity-related inflammation and metabolic syndrome-associated chronic diseases^15^,^16^. Metabolic modulations by SIRT1 has been demonstrated to protect against hepatic fat deposition in HFD fed mice^17^,^18^,^19^, in genetically-induced obese (ob/ob) mice^20^ and HFD -induced obesity model^21^. SIRT1 also involved in the modulation of hepatic bile acid homeostasis by regulating the expression of farnesoid X receptor (FXR) and SIRT1 deficiency in hepatocytes induced the formation of cholesterol gallstone^22^. Furthermore, SIRT1 attenuated hepatic inflammation by deacetylating nuclear factor kappa-light-chain-enhancer of activated B cells (NF-κB) p65, thereby decreasing the NF-κB induced expression of pro-inflammatory cytokines^18^,^23^. The activation of SIRT1 by caloric restriction and resveratrol had been shown to protect against development of fatty liver and metabolic diseases^24^,^25^.

MicroRNAs (miRNAs), the small non-protein-coding RNAs, play a critical role in the metabolic regulation^26^. Recent study suggested the involvement of miRNAs in the pathogenesis of hepatic steatosis, such as miR-122, miR-370 and miR-33^27^,^28^,^29^. There also have several miRs involved in the regulation of SIRT1 by binding to the 3′UTR of *Sirt1* mRNA then inhibiting SIRT1 protein expression^30^. Hepatic miR-34a is the only miR that related to both the expression of hepatic SIRT1 and the severity of NAFLD so far^31^,^32^.In addition, it has been shown that the elevated miR-34a in obesity reduces NAD^+^ levels and SIRT1 activity by directly targeting nicotinamide phosphoribosyltransferase (NAMPT), one of the rate-limiting enzyme for NAD biosynthesis^33^. Whether miR-34a/SIRT1 involved in the induction of steatosis mediated by HCD, in particular, if there is difference in miR-34a expression and SIRT1 protein level between isocaloric pair-fed HCD *vs*. HFD in mice is unknown.

In animal study, it is difficult to monitor food intake accurately using semi-purified powder diet due to food spilling into their plastic cages. Compared with semi-purified powder diet, the Lieber-DeCarli liquid diet conserved the advantage of having all the ingredients resolved in water and food intake is easily monitored. By group pair-feeding with the liquid diet, the energy intake, volume of the diets consumed and body weight gain of mice can be controlled among experimental groups^20^,^34^. In addition, the Lieber-DeCarli liquid diet can induce hepatic steatosis due to lacking of fiber. In the present study, we compared the effects of high-carbohydrate liquid diet (HCLD) on hepatic steatosis and inflammation with high-fat liquid diet (HFLD) in C57BL/6J mice by isocaloric-pair feeding, and the underlying mechanisms, especially the role of miR-34a and SIRT1 in hepatic steatosis development were explored.

## Results

### HCLD feeding induced more hepatic steatosis and higher liver weight and total cholesterol levels than HFLD

**Figure 1:**
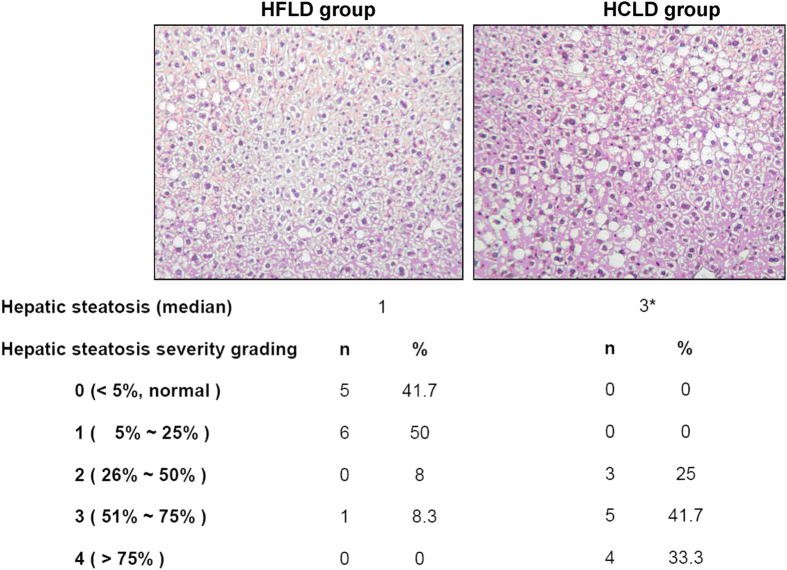
Hepatic steatosis treated with HCLD or HFLD for 16 weeks. Hepatic steatosis was assessed by hematoxylin and eosin (H&E) histopathological examination. Two independent investigators blinded to treatment groups examined the sections under light microscopy. The degree of steatosis was graded 0–4 based on the average percent of fat-accumulated hepatocytes per field at 100× magnification in 20 random fields, grading 0 ≤ 5% (normal liver), 1 = 5–25%, 2 = 26–50%, 3 = 51–75%, 4 ≥ 75%. The representative images of hepatic steatosis were presented. Non-parametric test was performed to compare the difference of the steatosis grading between two groups. ^*^indicated the significant difference between two groups, *P* < 0.05.

There was no statistical difference in the initial body weight, the final body weight, and body weight gain between HCLD and HFLD groups (Table 1). HCLD feeding resulted in significantly higher liver weights and the ratio of liver weight/body weight than HFLD feeding (*P* < 0.05, Table 1). Histological assessment of hepatic steatosis scores ranged from grade 2 to 4 with the median value of 3 in the HCLD treated group, and 75% (9/12) of the mice had hepatic steatosis with grade ≥ 3 and there had no mice with a steatosis grade <1. While in the HFLD treated mice, the steatosis scores ranged from grade 0 to 3 with the median value of 1, and only 8.33% (1/12) mice had hepatic steatosis with grade ≥3 and 11 out of 12 mice had the steatosis grade ≤1. The difference of steatosis grade was statistically significant between HCLD and HFLD treated groups (*P* < 0.001, Fig. 1). Using biochemical analysis, we did not detect any significant difference in hepatic TG levels, although the TG level was slightly higher in the HCLD fed mice, the change did not reach statistical significance as compared with that in the HFLD group (Table 1). HCLD feeding induced significantly higher TC deposition in liver comparing with HFLD treatment (Table 1, *P* < 0.01).

### HCLD feeding induced higher expression of hepatic miR-34a and lower levels of SIRT1 and NAMPT and phospho-AMPK

**Figure 2:**
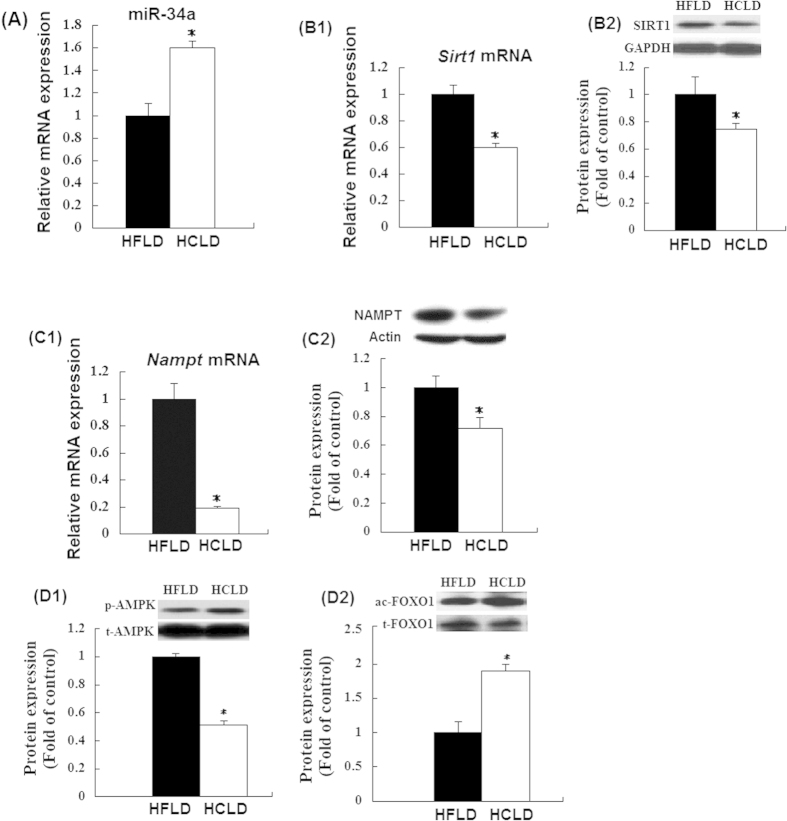
Relative expression of hepatic miR-34a, mRNA and protein levels of NAMPT and SIRT1, and proteins level of Ac-FOXO1 and AMPK. (**A**) Hepatic miR-34a was quantified by real-time quantitative PCR. U6 was used as controls; (**B–D**) mRNA and protein expression of SIRT1 NAMPT and SIRT1, and proteins level of Ac-FOXO1 and AMPK in liver tissue were determined by real-time quantitative PCR(mRNA level) and western blot (protein expression) respectively. Actin or GAPDH was used as controls. Values expressed as mean ± standard error of the mean (SEM), n = 12 for each group except for the determination of FOXO1 and AMPK, and sample number is 7 and 6 for HCLD group and HFLD respectively. t-test was performed to detect the difference between two groups. ^*^indicated the significant difference between two groups, *P* < 0.05.

As compared with HFLD feeding, HCLD feeding induced significantly higher expression of miR-34a in the livers (Fig. 2A). Since miR-34a binds to the 3′UTR of *Sirt1* mRNA and inhibits SIRT1 protein translation in liver^33^,^35^,we found that the levels of SIRT1 at both mRNA and protein were decreased in the livers of the mice treated with HCLD (Fig. 2B1,B2) indicating an inverse association between miR-34a and SIRT1 mRNA and protein levels. Meanwhile, we also detected the decreased mRNA expression and protein levels of *Nampt* in HCLD fed mice in contrast to HFLD fed mice (Fig. 2C1,C2). HCLD feeding decreased hepatic phospho-adenosine 5′-monophosphate (AMP)-activated protein kinase (AMPK) levels (Fig. 2D1) as compared with that of HFLD fed group, which has been shown to be positively correlated with the protein level and activity of SIRT1^36^. Furthermore, HCLD feeding increased protein expression of acetylated fork head box protein O1 (FOXO1), the target of SIRT1 mediated deacetylation (Fig. 2D2), indicating the decreased activity of SIRT1.

### HCLD feeding altered protein and mRNA expression of genes related to lipid and cholesterol metabolism

**Figure 3:**
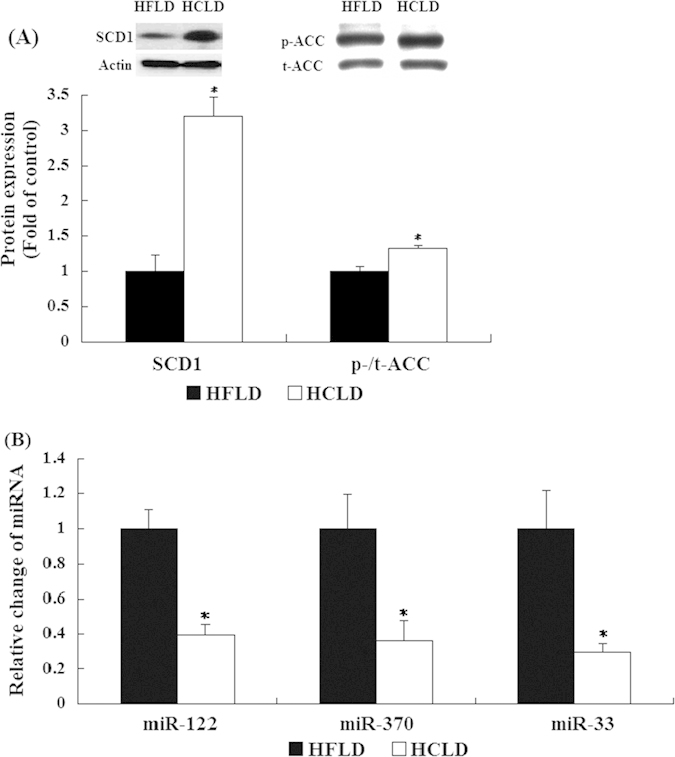
Proteins and microRNAs expression associated with lipid metabolism. (**A**) Proteins expression related to de no lipogenesis from liver tissue of HCLD and HFLD fed mice were determined by western blot. Actin was used as controls. (**B**) microRNAs expression of liver tissue related to lipid metabolism in mice fed with HCLD and HFLD was quantified by real-time quantitative PCR. U6 was used as controls. Values expressed as mean ± standard error of the mean (SEM), n = 12 for each group. t-test was performed to detect the difference between two groups. ^*^indicated the significant difference between two groups, *P* < 0.05.

**Figure 4:**
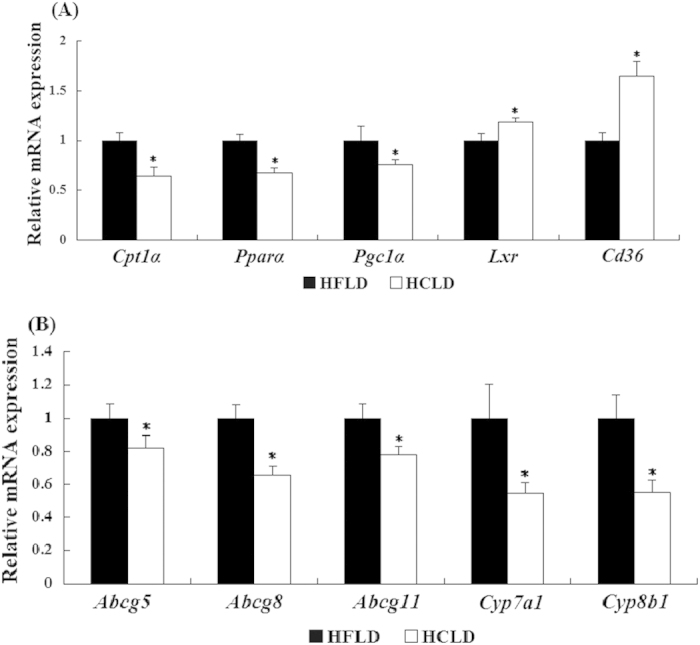
Relative mRNA expression of genes relating to lipid and cholesterol metabolism. mRNA expression of genes associated with fatty acid oxidation and transport (**A**) and cholesterol metabolism (**B**) from liver tissue of HCLD and HFLD fed mice were checked by quantitative real-time PCR. Values are mean ±standard error of the mean (SEM), n = 12 each group. Actin was used as controls, t-test was performed to detect the difference between two groups.^*^indicated the significant difference between two groups, *P* < 0.05.

Comparing to HFLD feeding, HCLD feeding (1) induced much higher expression of lipogenesis related proteins SCD1 and ACC (Fig. 3A). We examined both mRNA and protein levels of t-ACC and did not find any differences between the two groups; (2) significantly decreased expressions of miR-122 (61% decrease), miR-370 (64% decrease) and miR-33 (70% decrease), which involved in the regulation of lipid metabolism (Fig. 3B); 3) down-regulated the mRNA expression of genes related to fatty acid oxidation, such as carnitine palmytotransferase 1 (*Cpt1α*), peroxisome proliferator-activated receptor alpha (*Pparα*) and peroxisome proliferator-activated receptor-gamma coactivator-1 alpha (*Pgc1α*) (Fig. 4A); (4) resulted in higher mRNA expression of liver X receptor *α* (*Lxrα)* and fatty acid translocase (*Fat*/*Cd36*) (Fig. 4A); and 5) decreased mRNA expression of cholesterol efflux genes ATP-binding cassette sub-family G member 5 (*Abcg5)* and ATP-binding cassette sub-family G member 8 *(Abcg8)*, and bile acid synthesis genes ATP-binding cassette sub-family G member 11 *(Abcg11)*, cytochrome P450 (CYP)7A1*(Cyp7a1)* and *Cyp8b1* (Fig. 4B).

### HCLD feeding increased the incidence of inflammatory response and mRNA expression of inflammation related genes

**Figure 5:**
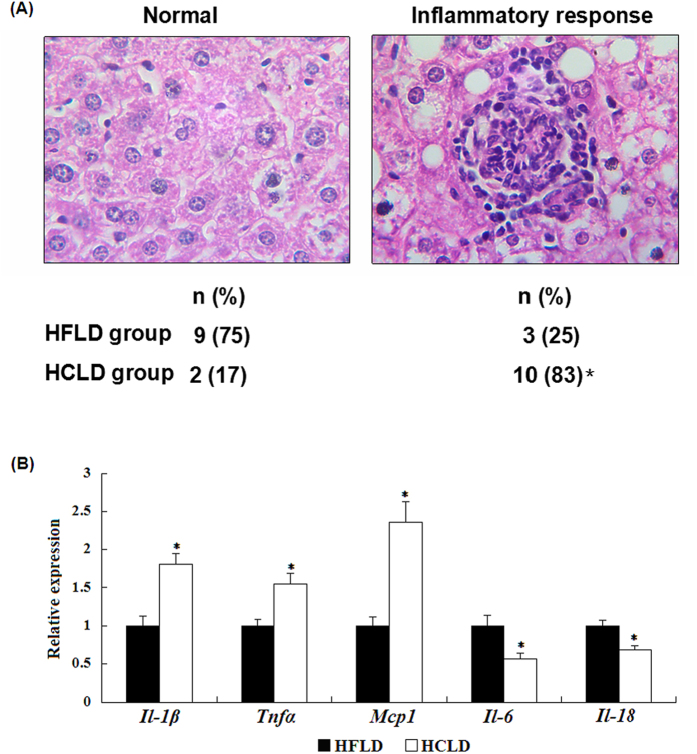
Hepatic inflammatory response (**A**) and relative mRNA expression of inflammatory cytokine genes of liver (B) after HCLD and HFLD treatment. (**A**) The incidence of hepatic inflammatory reaction was assessed by mononuclear inflammatory cells infiltration and inflammatory-cell clusters in 20 random fields at 100×magnification. The representative images of inflammatory response and normal were presented. X^2^-test was conducted to detect the difference of the incidence of inflammatory response between two groups. (**B**) mRNA expression of genes related to inflammation of liver tissue in mice treated with HCLD and HFLD was assessed by real-time quantitative PCR. Values expressed as mean ±standard error of the mean (SEM), n = 12 for each group. Actin was used as controls. t-test was performed on log transformed data for each treatment group. *indicated the significant difference between two groups, *P* < 0.05.

There was 83% (10/12) of mice that encountered positive inflammatory response in HCLD feeding group, while the incidence of inflammatory responses in HFLD treated group was only 25% (3/12), and the difference was statistically significant (Fig. 5A). Furthermore, HCLD feeding significantly increased the mRNA expression of pro-inflammatory cytokines interleukin-1β (*Il-1β*), tumor necrosis factor alpha (*Tnfα*) *and* monocyte chemotactic protein 1 (*Mcp1*), accompanying with decreased expression of interleukin-6 (*Il-6*) and interleukin-18 (*Il-18*) (Fig. 5B).

## Discussion

**Figure 6:**
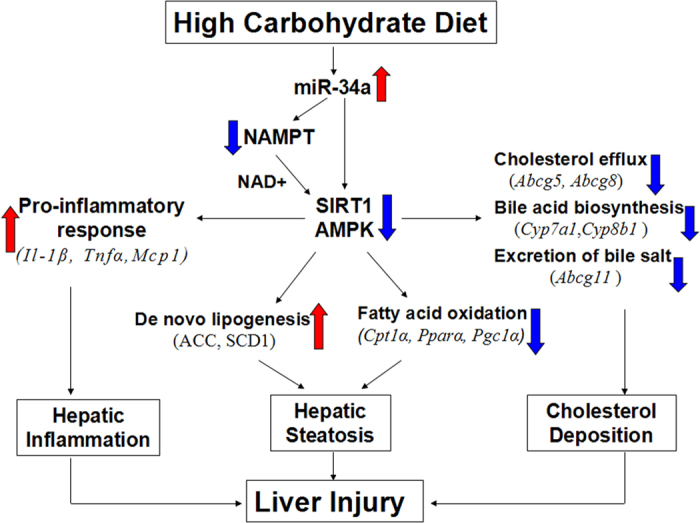
Proposed mechanisms of HCD induced liver injury in mice. High carbohydrate diet feeding upregualted the expression of miR-34a,which could inhibited the protein expression and activity of NAMPT, SIRT1 and AMPK, then increased the expression of genes related to hepatic inflammatory response, hepatic *de novo* lipogenesis and cholesterol deposition, decreased the expression of genes involved in fatty acid oxidation in the livers, thus, the enhanced hepatic inflammation, steatosis and cholesterol deposition synergesticlly resulted in the liver injury.

In the present study, despite similar energy intake and final body weight by group pair-feeding with the liquid diet, HCLD feeding for 16 weeks significantly increased the liver weight and the ratio of liver weight/body weight, and induced more severity of hepatic steatosis and cholesterol deposition than HFLD feeding. These changes were accompanied with significantly increased DNL related markers (SCD1 and ACC), and decreased mRNA level of genes related to fatty acid oxidation and cholesterol metabolism (Fig. 6). Although the difference in hepatic TG concentrations between HCLD and HFLD feeding mice did not reach statistical significance, this could be due to the fact that the distribution of steatosis in the liver is local, combined with the small portion of liver tissue used for biochemical analysis. We believe that the steatosis grading determined by histopathologic analysis for 20 random fields of the liver tissue section provided more reliable evidence for the hepatic lipid deposition. Meanwhile, HCLD feeding resulted in much higher incidence of hepatic inflammatory response accompanying with the increased expression of pro-inflammatory cytokines. Taken together, the present study provided strong experimental evidence that HCLD feeding, as compared with HFLD feeding, induced more detrimental effects in the liver.

The important finding in the present study was that HCLD feeding induced steatosis potentially by miR-34a/SIRT1 axis. Previous studies supported the involvement of SIRT1 in HFD induced hepatic steatosis by deacetylating the proteins regulating lipogenesis^37^ and fatty acid oxidation^17^,^18^,^19^,^38^. The increased expression of SIRT1 could promote the deacetylation of sterol response element-binding protein 1c (SREBP1c) and inhibit its activity, which subsequently down-regulated the expression of genes associated with lipogenesis, such as ACC and SCD1^39^. In our present study, the decreased protein level of SIRT1 and its activity, as evidenced by increased acylated-FOXO1, resulted in the increased proteins level of ACC and SCD1, which supported the role of SIRT1 in mediating hepatic steatosis induced by HCLD feeding. Interestingly, we observed that the HCLD feeding resulted in higher expression of miR-34a as compared with that in HFLD fed group, and recent studies showed that miR-34a could inhibit SIRT1 expression by directly binding to the 3′UTRs of its mRNAs and thus, effectively decreased the protein expression and inhibited the activity of SIRT1^35^,^40^. Indeed, we detected the decreased mRNA expression and protein levels of Sirt1 and Nampt in liver after HCLD feeding. As suggested by Gao *et al.*^41^, once there had a precise binding to the 3′UTR of target mRNA, the microRNA could inhibit target protein production by decreasing target mRNA level, thus, our observation lead us to speculate that HLCD induced miR34a which binds to the 3′UTR of mRNAs of Sirt1 and Nampt could block the SIRT1/NAMPT protein translation by destabilization of target mRNAs expression of both Sirt1 and Nampt genes. Therefore, the higher expression of miR-34a in HCLD feeding might account for the decreased protein levels of SIRT1 and NAMPT, as well as SIRT1 activity (Fig. 6). However, the mechanisms how HCLD affected miR-34a expression is unclear.

Although there only had 26% decrease of hepatic SIRT1 protein level after HCLD feeding in contrast to HFLD fed mice, considering the study was conducted *in vivo* for 4 months, we believe that the extent of reduction in hepatic SIRT1 at protein levels in HCLD fed mice compared to HFLD fed mice is significant, because such chronic reduction of SIRT1 will contribute significantly to the long term impact. In addition, the more severe steatosis induced by HCLD is correlated with the levels of the induction of miR-34a, the reduction of SIRT1 protein levels and its activity (as showed by upregulated protein level of ac-FOXO1, the target of SIRT1’s deaceylated acitivity), as well as the reduction of NAMPT. All of these changes can greatly promote fatty liver development in HCLD fed mice.

Furthermore, we observed that phosphorylation of AMPK decreased after HCLD feeding. We did not detect any differences on the totals of AMPK and FOXO1, but p-AMPK and ac-FOXO1, which indicated that the down-regulated activity of AMPK (phospharylation) and SIRT1 (deacetylation) induced by HCLD contributed greatly to fatty liver development. It has been reported that the activation of AMPK could increase oxidative metabolism by up-regulating SIRT1 activity^36^ and the suppressed activation of AMPK inhibited the deacetylated activity of SIRT1 on its targets, and ultimately inhibited fatty acid oxidation^39^,^42^. Indeed, the decreased mRNA expressions of fatty acid oxidation related genes *Cpt1*, *Ppara* and *Pgc1a* also supported the involvement of SIRT1/AMPK in the decreased fatty acid oxidation, and subsequently promoted the steatosis mediated by HCLD feeding. We further observed that HCLD feeding up-regulated both mRNA expressions of *Lxrα and Cd36.* Since SIRT1 down-regulates the expression of CD36 by inhibiting the activity of LXR^43^, thus, it is possible that the decreased SIRT1 induced by HCLD could increase the mRNA expression of *Cd36*, which promoted fatty acid availability to the liver and facilitated much more synthesis of TG and cholesterol ester, and ultimately contributed to more serious hepatic steatosis. This notion was supported by previous study that CD36 knock-out mice exhibited much higher level of fatty acid and TG in circulation as the decreased transport of free fatty acids to the livers^44^.

Cholesterol efflux, intestinal cholesterol absorption and cholesterol transformation into bile acids in the liver synergistically maintained the balance of cholesterol metabolism^45^,^46^. Recent research work suggested that SIRT1 is required for bile acid absorption and systemic bile acid homeostasis in mice^47^. In the present study, we observed that the mRNA expressions of *Cyp7a1* and *Cyp8b1* genes involved in bile acid biosynthesis, *Abcg5* and *Abcg8* genes involved in cholesterol efflux, and *Abcg11* gene involved in the excretion of bile salt in the liver were all decreased by HCLD feeding. These changes could promote the cholesterol deposition in liver, which were in consistent with the result of the higher hepatic TC levels after HCLD feeding. Therefore, it is possible that the decreased SIRT1 mediated by HCLD feeding promoted the TC deposition in liver by inhibiting bile acid biosynthesis, cholesterol efflux and excretion of bile salt.

In the present study, we found the significantly decreased expression of miR-122, miR-370 and miR-33 after HCLD treatment, which may provide an additional mechanism regarding the hepatic steatosis induced by HCLD. It has been shown that miR-122, miR-370 and miR-33 are hepatocyte specific microRNAs, and involve in the regulation of hepatic lipid metabolism, cholesterol and fatty acid synthesis by negative regulation of their target genes^27^,^28^,^29^. The decreased expression of miR-122 up-regulated the level of genes associated with triglycerides biosynthesis and storage, subsequent microsteatosis and liver inflammation^48^. The effects of miR-370 on fatty acids and hepatic TG accumulation might be through the modulation of miR-122 expression, as miR-370 up-regulates the expression of miR-122^49^. The inhibition of miR-33 increased the lipid accumulation in the liver of mice which mediated by the increased expression of genes, such as ACC, in the livers^50^. Our present data showed the significantly decreased expression of miR-122, miR-370 and miR-33, accompanied with the increased expression of lipogenetic proteins ACC and SCD1 induced by HCLD feeding, supported the role of those miRNAs in the pathogenesis of hepatic steatosis.

Previous studies have documented that excess lipid and higher cholesterol deposition in the liver could induce liver injury mediated by oxidative stress and the inflammation^51^. SIRT1 plays an anti-inflammation role mainly by deacetylating NF-κB and decreasing the expression of pro-inflammatory cytokines. Indeed, in the present study, the increased mRNA of inflammatory genes *Tnfα, Mcp1* and *Il-1β*, as well as increased hepatic inflammatory foci and inflammation cell infiltration could be due to the down-regulated expression of SIRT1 in HCLD feeding mice. Unexpectedly, we found the lower expression of *Il-6* in HCLD fed mice. Since it has been reported that IL-6 conserved both anti-inflammatory and pro-inflammatory effects, and its anti-inflammatory role was accompanied with the inhibition of TNFa^52^, therefore, the lower mRNA expression of *Il-6* combined with higher mRNA level of *Tnfa* could contribute to the inflammatory response induced by HCLD. The role of IL-18 in mediating hepatic inflammation is conflicting. For example, one previous study showed that the expression level of IL-18 was higher in the rat liver of NAFLD group than in the control group^53^, but the circulating IL-18 level was not altered in male subjects with NAFLD^54^. As showed in our study, HCLD feeding decreased hepatic expression of *Il-18* in the livers, which was in accordance with the previous study that IL-18 negatively related to hepatic steatosis and inflammation^55^. These discrepancies might be due to the different source of tissue and experimental subjects.

The limitations of the study were that we only focused on whether isocaloric HCLD feeding led to more fatty liver through the involvement of hepatic miR-34a/SIRT1 in mice. Both liver weight and liver/body ratio were significantly larger in HCLD *vs*. HFLD, which support our notion that more fatty liver induced by HCLD could be due to increased hepatic DNL and decreased fatty acid oxidation by miR-34a/SIRT1 regulation. However, both body weight and body weight gain in HCLD group are slightly greater than those in HFLD group, although they did not reach statistically different. Therefore, potential systemic alteration (e.g., plasma levels of free fatty acid, TG, glucose and insulin) and other metabolic factors (e.g., body composition, adiponection) involved in hepatic steatosis should be investigated. Indeed, our recent study has shown that the up-regulation PPARα and PPARγ and their associated genes induced by tomato carotenoids in mesenteric fat tissue had promoted fatty acid utilization, and subsequently reduced lipid delivery from adipose tissue to the liver^56^ and lycopene can modulate plasma adiponectin concentration and mRNA levels of adiponectin, SIRT1 and FoxO1 in adipose tissue of obese rats^57^.

In summary, the present study (Fig. 6) demonstrated that the isocaloric HCD feeding induced greater severity in hepatic steatosis and inflammatory response than HFD feeding, which were associated with higher expression of miR-34a, down-regulated protein level and activity of SIRT1, and suppressed activation of AMPK. All those changes resulted in subsequent promotion of DNL, inhibition of fatty acid oxidation and cholesterol metabolism. Thus, targeting miR-34a/SIRT1 axis might be an effective strategy to alleviate the deleterious effects of HCD on liver.

## Methods

### Animals and animal treatment

Male C57BL/6J mice (6 weeks old) from the Jackson Laboratory were individually housed in plastic cages under the standard conditions of temperature and humidity with 12 h light-dark cycles. The animal protocol for the study was approved by the Institutional Animal Care and Use Committee at the USDA Human Nutrition Research Center on Aging at Tufts University, and the methods were carried out in accordance with the approved guidelines.

After one week of acclimation, mice were randomly divided into two groups (n = 12 per group), and given two different types of liquid diet for 16 weeks: 1) the Lieber-DeCarli high-fat, low-carbohydrate liquid diet (HFLD, 60% of total energy derived from fat, 22% from carbohydrate, and 18% from protein, No.712037), 2) the Lieber-DeCarli high-carbohydrate, low-fat liquid diet (HCLD, 12% of total energy derived from fat, 70% from carbohydrate, and 18% from protein, No. 710028), which were purchased from Dyets, Inc. The ingredients of those two diets were displayed in Table 2. Maltose dextrin is the major source of carbohydrate with a high glycemic index, and corn oil provides as a major source of fat. Both diets provide 1.0 Kcal energy per ml diet. To achieve isocaloric intake, mice were group-pair fed during the period of the study, as described^20^. The amount of diet fed to the HCLD group was based on the mean consumption of diet by the HFLD group from the preceding day. The diets were prepared twice a week and were stored at 4 °C in opaque bottles. Body weight was recorded weekly. At the end of the experiment, the mice were euthanized, livers were removed, weighed, and parts of the liver were stored at −80 °C for western blot and RT-PCR analysis, or fixed in 10% formalin solution to conduct histological analysis.

### Histopathological evaluation of liver lesions

Five μm sections of formalin-fixed, paraffin-embedded liver tissue were stained with hematoxylin and eosin (H&E) for histopathological examination. Two independent investigators blinded to treatment groups examined the sections under light microscopy. Liver histopathology areas were graded according to steatosis magnitude (both macro- and micro-vesicular) as described previously^9^. Briefly, the degree of steatosis was graded 0–4 based on the average percent of fat-accumulated hepatocytes per field at 100× magnification in 20 random fields, grading 0 ≤5% (normal liver), 1 = 5–25%, 2 = 26–50%, 3 = 51–75%, 4 ≥75%. Positive incidence of inflammatory response was assessed according to both mononuclear inflammatory cells infiltration and number of inflammatory-cell clusters in 20 random fields at 100× magnification, as described^9^. A ZEISS microscope with a PixeLINK USB 2.0 (PL-B623CU) digital Camera and Pixe LINK μScope Microscopy Software was used for image capture of all histological examinations.

### Determination of hepatic cholesterol and triglycerides (TG) content

Hepatic tissue (50 mg wet weight) was used to determine the total cholesterol (TC) content by using a Total Cholesterol Assay Kit (Colorimetric) (STA-384, CELL BIOLABS, INC) and according to the manufacture’s protocol. Hepatic total TG contents were determined according to previously described^58^.

### RNA extraction and quantitative real-time PCR

RNA extraction of liver and quantitative real-time PCR were conducted as previously described^9^. For microRNA quantification, cDNA was synthesized with Moloney murine leukemia virus RT (Invitrogen) according to the manufacturer’s recommendations, and the mRNA level was normalized to U6. The mRNA levels were determined using the ^△△^C_T_ method and expressed as a fold of the control group.

### Protein extraction of hepatic whole cell lysate and western blot analysis

Protein extraction of liver and western blotting were performed as previously described^21^. The protein concentration of the whole-tissue extracts was determined by Bradford assay (Bio-Rad, CA, USA). Primary antibodies included SIRT1, Acetylated-, total-FOXO1 (Ac-FOXO1, t-FOXO1) were all purchased from Santa Cruz Biotechnology (Santa Cruz, TX, USA). The antibodies against phospho- and total- AMPK (p-AMPK, t-AMPK), SCD1, phospho- and total-ACC were all purchased from Cell Signal Biotechnology (Cell Signaling, MA, USA). The antibody against NAMPT was purchased from Abcam (Abcam, Cambridge, UK). The secondary antibodies included horseradish peroxidase-conjugated anti-rabbit, anti-mouse antibodies (Bio-Rad, CA, USA). β-actin was used as equal loading control of proteins (Sigma-Aldrich). GS-710 Calibrated Imaging Densitometer (Bio-Rad) was used to quantify the intensities of protein bands.

### Statistical analysis

Results were expressed as mean ± standard deviation (SD) for animal weights, liver weight, liver weight/body weight and inflammatory foci. Hepatic cholesterol content, mRNA, miRNAs and protein levels were expressed as mean ± standard error of the mean (SEM). Data from 2 groups were compared using Student’s t-test. Steatosis grading is presented as median (grading range). The ratio of liver weight/body weight and steatosis grading or data with non-normal distribution were tested by non-parametric test. Comparison of the incidences of inflammation between the two groups was conducted by χ^2^ test. All data were analyzed using SPSS software. *P* < 0.05 was considered significant.

## Additional Information

**How to cite this article**: Li, X. *et al.* Isocaloric Pair-Fed High-Carbohydrate Diet Induced More Hepatic Steatosis and Inflammation than High-Fat Diet Mediated by miR-34a/SIRT1 Axis in Mice. *Sci. Rep.* **5**, 16774; doi: 10.1038/srep16774 (2015).

## Figures and Tables

**Table 1:** Study outcomes.

Index	HFLD	HCLD
Animal number	12	12
Initial body weight (g)	18.9 ± 1.7	18.9 ± 1.6
Final body weight (g)	34.6 ± 5.6	38.9 ± 3.6
Body weight gain (g)	15.7 ± 4.6	19.9 ± 3.1
Liver weight (g)	1.4 ± 0.3	2.4 ± 0.7*
Liver weight /body weight (%)	3.9 ± 0.5	6.1 ± 1.3*
Hepatic total cholesterol (mg/g)	1.4 ± 0.3	1.6 ± 0.3*
Hepatic triglycerols (mg/g protein)	366.6 ± 16.5	397.8 ± 48.8

Data are presented as mean ±standard deviation (SD). For given row, *indicated the significant difference between two groups, with *P* < 0.05 Abbreviations: HFLD: high fat liquid diet, HCLD: high carbohydrate liquid diet.

**Table 2:** Ingredient of High-fat, Low-carbohydrate diet (HFLD, No. 712037) and High-carbohydrate, Low-fat diet (HCLD, No. 710028).

Ingredient	HFLD (710028)		HCLD (712037)	
	Grams/kg	Kcal/L	Grams/kg	Kcal/L
Casein(100 Mesh)	41.4	176.778	41.4	176.778
L-Cystine	0.5	2	0.5	2
DL-Methionine	0.3	1.2	0.3	1.2
Corn Oil	56	495.04	2.5	22.1
Olive Oil	8.4	74.256	8.4	74.256
Safflower Oil	2.7	23.868	2.7	23.868
Maltose Dextrin	54	213.84	173.2	685.872
Cellulose	10	0	10	0
Mineral Mix	8.75	4.1125	8.75	4.1125
Vitamin Mix	2.5	9.5	2.5	9.5
Choline Bitartrate	0.53	0	0.53	0
Xanthan Gum	3	0	3	0
